# Evaluation of the Truncated Cone–Rhomboid Pyramid Formula for Simplified Right Ventricular Quantification: A Cardiac Magnetic Resonance Study

**DOI:** 10.3390/jcm13102850

**Published:** 2024-05-12

**Authors:** Annemarie Kirschfink, Michael Frick, Ghazi Al Ateah, Kinan Kneizeh, Anas Alnaimi, Rosalia Dettori, Katharina Schuett, Nikolaus Marx, Ertunc Altiok

**Affiliations:** 1 Department of Cardiology, Angiology and Intensive Care, University Hospital, RWTH Aachen University, Pauwelsstrasse 30, 52074 Aachen, Germany; 2 Department of Cardiology, Nephrology and Internal Intensive Care Medicine, Rhein-Maas Klinikum, Mauerfeldchen 25, 52146 Wuerselen, Germany

**Keywords:** cardiac magnetic resonance, right ventricular volume, right ventricular function

## Abstract

**Background/Objective:** Cardiac magnetic resonance (CMR) is the reference method for right ventricular (RV) volume and function analysis, but time-consuming manual segmentation and corrections of imperfect automatic segmentations are needed. This study sought to evaluate the applicability of an echocardiographically established truncated cone–rhomboid pyramid formula (CPF) for simplified RV quantification using CMR. **Methods**: A total of 70 consecutive patients assigned to RV analysis using CMR were included. As standard method, the manual contouring of RV-short axis planes was performed for the measurement of end-diastolic volume (EDV) and end-systolic volume (ESV). Additionally, two linear measurements in four-chamber views were obtained in systole and diastole: basal diameters at the level of tricuspid valve (Dd and Ds) and baso-apical lengths from the center of tricuspid valve to the RV apex (Ld and Ls) were measured for the calculation of RV-EDV = 1.21 × Dd^2^ × Ld and RV-ESV = 1.21 × Ds ^2^ × Ls using CPF. **Results:** RV volumes using CPF were slightly higher than those using standard CMR analysis (RV-EDV index: 86.2 ± 29.4 mL/m^2^ and RV-ESV index: 51.5 ± 22.5 mL/m^2^ vs. RV-EDV index: 81.7 ± 24.1 mL/m^2^ and RV-ESV index: 44.5 ± 23.2 mL/m^2^) and RV-EF was lower (RV-EF: 41.1 ± 13.5% vs. 48.4 ± 13.7%). Both methods had a strong correlation of RV volumes (ΔRV-EDV index = −4.5 ± 19.0 mL/m^2^; r = 0.765, *p* < 0.0001; ΔRV-ESV index = −7.0 ± 14.4 mL/m^2^; r = 0.801, *p* < 0.0001). **Conclusions:** Calculations of RV volumes and function using CPF assuming the geometrical model of a truncated cone–rhomboid pyramid anatomy of RV is feasible, with a strong correlation to measurements using standard CMR analysis, and only two systolic and diastolic linear measurements in four-chamber views are needed.

## 1. Introduction

The dysfunction of the right ventricle (RV) is associated with increased cardiovascular morbidity and mortality in congenital and acquired heart disease [1]. Therefore, RV quantification is important in everyday clinical practice even though RV assessment is still challenging due to its complex anatomy. Standard echocardiographic approaches are often limited in providing correct and reproducible results [2]. Therefore, Al Ateah et al. (2023) have established a formula based on a truncated cone and rhomboid pyramid model of the RV and performed only two linear measurements in four-chamber view for the echocardiographic calculation of RV volumes and function. Echocardiographic RV volumes and function using the cone–pyramid formula (CPF) highly agreed with those measured using cardiac magnetic resonance (CMR). The echocardiographic RV analysis using the CPF method was more accurate in detecting reduced RV function than standard echocardiographic parameters like tricuspid annular plane systolic excursion (TAPSE) or RV fractional area change (FAC) with CMR as the reference method [3].

CMR is the current gold standard for RV analysis [4]. Even for CMR specialists, RV quantification through the manual contouring of endocardial borders on a stack of short-axis cine sequences with full coverage of the RV is time-consuming and susceptible to observer variability [5,6]. Although semi- and fully automatic segmentation techniques are increasingly used, these methods are not available everywhere, manual corrections of incorrect segmentations have to be performed, and a certain amount of reader experience is required [7]. Therefore, a rapid and simple method is needed as a screening tool for RV analysis.

The aim of this study was to evaluate the applicability of a recently established echocardiographic method to CMR. This method is based on a truncated cone and rhomboid pyramid model of the RV and only requires two linear measurements in end-diastolic and end-systolic four-chamber views for the calculation of RV volumes and function.

## 2. Materials and Methods

In this retrospective single-center study, 70 consecutive patients (56 ± 15 years; 49 male) were included, who were assigned to RV analysis using CMR for different indications. This study was approved by the Institutional Ethics Committee of the University Hospital of RWTH Aachen (EK 012/21) and the need for consent was waived. This study was performed according to the guidelines of the Declaration of Helsinki.

### 2.1. CMR Protocol

CMR examinations were performed on a 1.5 Tesla MR-scanner (Philips Achieva, Philips Healthcare, Best, The Netherlands) using a 32-element cardiac coil or a 5-element cardiac synergy coil for signal reception. Standard cine steady-state free precession (SSFP) sequences in 3 long-axis geometries (2-, 3-, and 4 chamber view) as well as a contiguous stack of short-axis images (slice thickness: 8 mm) covering the entire ventricles were acquired.

As standard method, measurements of RV end-diastolic volume (RV-EDV) and RV end-systolic volume (RV-ESV) were obtained by manually contouring endocardial borders in end-diastole and end-systole on each slice using the disk summation method. Papillary muscles and trabeculations were included in RV volumes. For a better delineation of the base of the heart and the tricuspid valve, short-axis slices were correlated to the corresponding 4-chamber view.

**Figure 1:**
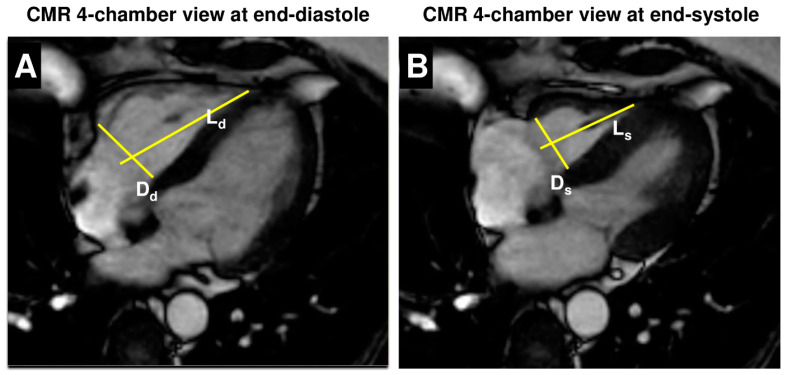
Linear measurements in 4-chamber view for quantification of RV volumes and function using the cone–pyramid formula (CPF). In 4-chamber view at end-diastole (**A**) and end-systole (**B**), basal diameters at the level of the tricuspid valve (Dd and Ds) and baso-apical length from the center of the tricuspid valve to the RV apex (Ld and Ls) were measured for the calculation of RV size and function using the cone–pyramid formula (CPF).

The proposed CPF assumes a simplified RV geometry of a truncated cone minus a truncated rhomboid pyramid resulting in calculations of volumes based on this assumption [3]. In the 4-chamber views, two linear measurements were performed: (1) RV basal diameters were obtained at the level of the tricuspid valve in end-diastole (Dd) and in end-systole (Ds) for the axial function of RV, and (2) the RV baso-apical length from the center of the tricuspid valve to the RV apex in end-diastole (Ld) and in end-systole (Ls) for longitudinal RV function (Figure 1).

These 4-chamber view measurements were inserted in the cone–pyramid formula which has been previously described for the echocardiographic RV assessment of RV end-diastolic and end-systolic volume: RV-EDV = 1.21 × Dd^2^ × Ld; and RV-ESV = 1.21 × Ds^2^ × Ls [3]. RV volumes were indexed to body surface area (BSA). For both methods, RV ejection fraction (RV-EF) was determined as follows: RV-EF = (RV-EDV − RV-ESV) × 100/RV-EDV. RV volumes and diameters were analyzed by experienced, board-certified CMR cardiologists.

According to published CMR reference values, enlarged RV was defined as RV-EDV index > 123 mL/m^2^ in men and >104 mL/m^2^ in women or RV-ESV index > 59 mL/m^2^ in men and >48 mL/m^2^ in women. RV ejection fraction < 42% in men and <46% in women was classified as reduced RV function [8].

Calculations of RV volumes (RV-EDV, RV-ESV) and RV function (RV-EF) using the CPF method were repeated in 30 randomly selected patients by the initial observer 1 at least 6 months after the first analysis and independently by a second observer 2 to determine intra- and interobserver variability.

### 2.2. Statistical Analysis

Categorical variables were indicated as count (%) and continuous variables as mean ± standard deviation. Correlations of CMR standard measurements and CPF-based CMR measurements were evaluated. Scatter diagrams with regression line display the concordance between the calculations of standard CMR analysis and the CPF method, and the agreement between the two methods was analyzed using Bland–Altman analyses. Receiver-operating characteristics (ROC) curve analyses including area under the curve (AUC) and the corresponding 95%-confidence interval (CI) were used to identify patients with enlarged RV and reduced RV function using CPF. Sensitivity and specificity are shown for cut-off values with the highest Youden index. Intra- and interobserver variability were evaluated by intraclass correlation coefficient with 95%-CI. Version 13.0 of the MedCalc statistical software (MedCalc Software Ltd., Ostend, Belgium) was used for statistical analyses. Statistical significance was indicated by *p*-values less than 0.05.

## 3. Results

In all 70 (100%) patients, linear RV measurements in CMR four-chamber views and the assessment of RV volumes (RV-EDV and RV-ESV) and function (RV-EF) using the cone–pyramid formula were feasible. Table 1 shows patients’ clinical characteristics and their left and right ventricular volumes and function measured using the CMR standard method. Cardiomyopathy of various etiologies was present in 31 (44%) of all patients: 17 (24%) patients presented with a dilatative cardiomyopathy, 5 (7%) patients with an ischemic cardiomyopathy, 2 (3%) with a toxic cardiomyopathy, and 1 patient each suffered from an obstructive hypertrophic cardiomyopathy, non-compaction cardiomyopathy, valvular cardiomyopathy, septic cardiomyopathy, sarcoidosis, amyloidosis, and takotsubo syndrome.

According to CMR reference values [8], enlarged RV by the RV-EDV index was present in 7 (10%) patients and by the RV-ESV index in 19 (27%) patients. Accordingly, in 22 (31%) patients, RV-EF was reduced, indicating impaired RV function.

### 3.1. Comparison of RV Volumes and Function

**Figure 2:**
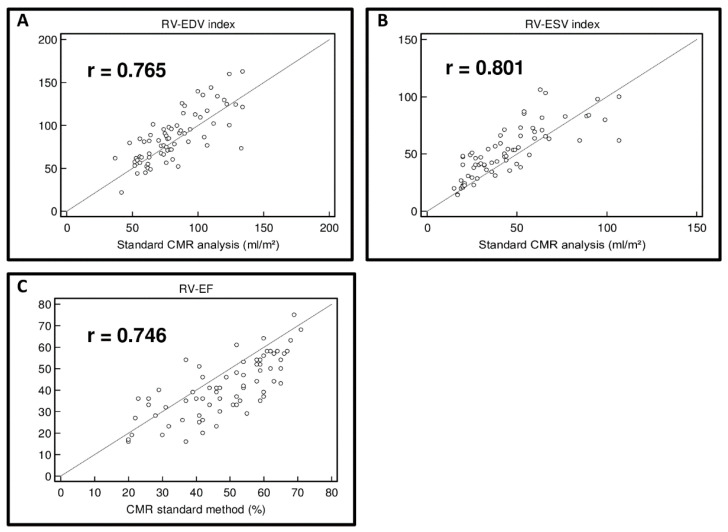
Correlation of RV volumes and RV function using the cone–pyramid formula (CPF) and using standard CMR analysis. Scatter diagram of right ventricular end-diastolic volume (RV-EDV) index (**A**), right ventricular end-systolic volume (RV-ESV) index (**B**), and right ventricular ejection fraction (RV-EF) (**C**) using the cone–pyramid formula (CPF) compared to standard CMR analysis.

**Figure 3:**
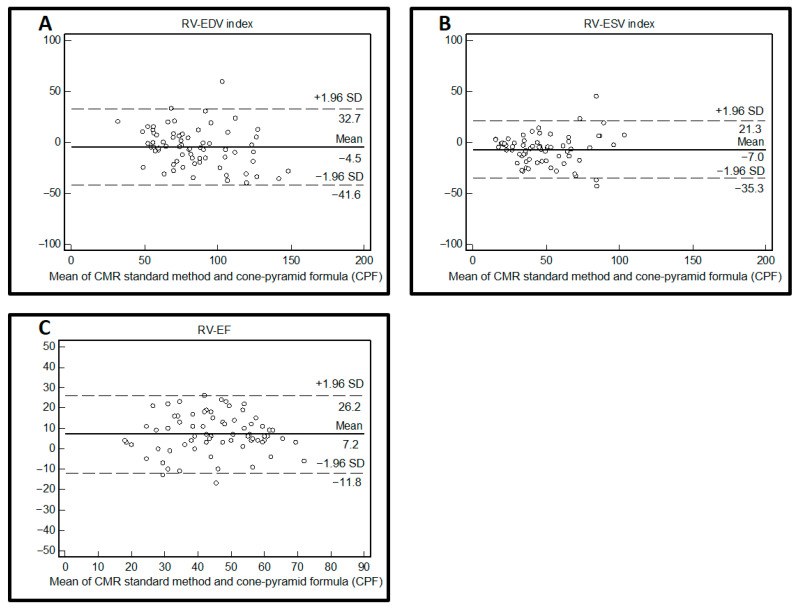
Agreement of RV volumes and RV function using the cone-pyramid formula (CPF) and standard CMR analysis. Bland–Altman plot for the method comparison of measurements of right ventricular end-diastolic volume (RV-EDV) index (**A**), right ventricular end-systolic volume (RV-ESV) index (**B**), and right ventricular ejection fraction (RV-EF) (**C**) calculated using the cone–pyramid formula (CPF) compared to standard CMR analysis.

RV volumes using the CPF method were slightly higher than RV volumes using standard CMR measurements: the RV-EDV index was calculated as 86.2 ± 29.4 mL/m^2^ and the RV-ESV index as 51.5 ± 22.5 mL/m^2^ using CPF, whereas standard CMR analysis resulted in a RV-EDV index of 81.7 ± 24.1 mL/m^2^ and RV-ESV index of 44.5 ± 23.2 mL/m^2^. RV-EF using CPF was lower in comparison to standard CMR analysis (RV-EF: 41.1 ± 13.5% vs. 48.4 ± 13.7%). Both methods showed a strong correlation of RV volumes (ΔRV-EDV index = −4.5 ± 19.0 mL/m^2^; correlation coefficient r = 0.765, *p* < 0.0001; ΔRV-ESV index = −7.0 ± 14.4 mL/m^2^; correlation coefficient r = 0.801, *p* < 0.0001) (Table 2, Figure 2 and Figure 3). Even though lower RV-EF values were calculated using the CPF method, the correlation of RV-EF between the CPF method and standard CMR measurements was still good (ΔRV-EF = 7.2 ± 9.6%; correlation coefficient r = 0.746, *p* < 0.0001) (Table 2, Figure 2 and Figure 3).

### 3.2. Identification of Patients with Enlarged RV Volumes and Reduced RV Function

**Figure 4:**
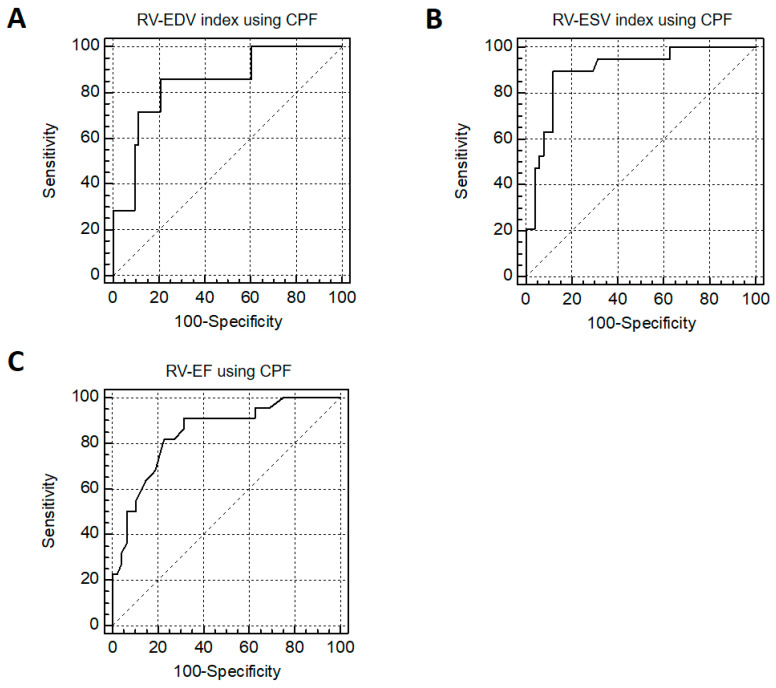
Identification of patients with enlarged RV size and reduced RV function. Receiver-operating characteristics (ROC) analysis for the prediction of enlarged right ventricular volumes (RV-EDV index (**A**) and RV-ESV index (**B**)) and reduced RV function (RV-EF (**C**)) using the cone–pyramid formula (CPF) and as defined by CMR reference values based on Kawel-Boehm et al. (2020) [8].

Patients with enlarged RV-EDV indexed by BSA (*n* = 7) and enlarged RV-ESV indexed by BSA (*n* = 19), defined by CMR reference values based on Kawel-Boehm et al. (2020), were identified with high accuracy using the CPF method (RV-EDV index: AUC = 0.841, 95%-CI 0.770 to 0.902; cut-off value: >99.4 mL/m^2^; sensitivity = 85.7%; specificity = 79.4%; RV-ESV index: AUC = 0.898, 95%-CI 0.803 to 0.958; cut-off value: >59.3 mL/m^2^; sensitivity = 89.5%; specificity = 88.2%) (Figure 4) [8]. Likewise, the identification of reduced RV function (*n* = 22) showed high accuracy (RV-EF: AUC = 0.845, 95%-CI 0.739 to 0.921; cut-off value: ≤40%; sensitivity = 90.9%; specificity = 68.8%) (Figure 4).

### 3.3. Intra- and Interobserver Variability

Table 3 shows the intra- and interobserver variability of the CPF method in 30 randomly selected patients re-analyzed by the initial observer (Observer 1) and a second observer (Observer 2). The intraobserver and interobserver agreement of RV volumes was high and the differences between the measured values were small (intraobserver agreement: ΔRV-EDV = 0.6 ± 6.4 mL, ICC = 0.9949; ΔRV-ESV = −1.7 ± 4.1 mL, ICC = 0.9959; interobserver agreement: ΔRV-EDV = 12.9 ± 16.9 mL, ICC = 0.9650; ΔRV-ESV = −8.5 ± 12.6 mL, ICC = 0.9574). The intraobserver agreement of RV-EF was still high while the agreement of the calculations of RV-EF was reasonable between the two observers (intraobserver agreement: ΔRV-EF = 1 ± 3.2%, ICC = 0.9684; interobserver agreement: ΔRV-EF = 10 ± 7.9%, ICC = 0.7982).

## 4. Discussion

The major findings of this study were as follows: (1) the application of the CPF method on CMR images with only two linear measurements in 4-chamber views was feasible in all patients; (2) RV volumes and function using the CPF method based on the geometrical assumption of a truncated cone and rhomboid pyramid model showed a strong correlation to measurements using standard CMR analysis; and (3) the CPF method identified patients with enlarged RV volumes and reduced RV function with high accuracy, with the definition of reference values by the CMR standard method [8].

CMR is the gold standard for the non-invasive imaging of cardiac chamber quantification, with an increasing availability in daily clinical routine [9]. Impaired RV function is known to be associated with increased cardiovascular morbidity and mortality in various heart diseases [1]. Moreover, RV dysfunction in CMR analysis is of prognostic value in patients with diagnosed and suspected cardiovascular disease or in patients with heart failure [10,11,12]. The standard CMR analysis of RV is performed by manually contouring end-diastolic and end-systolic endocardial borders on each slice of a contiguous stack of short-axis slices, which visualize the whole RV from the apex to the base and its partly complex geometry from apex to RV base [9]. The manual contouring of the RV is time-consuming and less reproducible than contouring the left ventricle (LV) [6]. Especially the contouring of the last two basal short-axis slices can be challenging because of the through plane motion of the tricuspid valve caused by the longitudinal RV shortening during systole, and may lead to inaccurate segmentation and observer variability [13,14]. For a better identification of the tricuspid valve as the border of the basal RV, a comparison to long-axis views like the four-chamber view and axial views has been recommended [9,14]. In recent years, semi- and fully automatic segmentation methods have been developed for LV and RV quantification using artificial intelligence: on the one hand, the time for contouring endocardial borders is considerably reduced, but on the other hand, manual corrections of incorrect segmentations are more often necessary for the right than the left ventricle. Besides human atypical errors, the contouring of the most basal slice but also the most apical slice tends to be inaccurate when using automatic segmentation methods and needs to be corrected, which may be challenging and error-prone, especially for unexperienced readers [6,7,9,14,15].

Measurements for RV quantification using the CPF method are performed without additional software tools on standard four-chamber views, which is routinely obtained according to the recent recommendations for standardized CMR protocols [16]. Since only two linear measurements in systole and diastole are needed, CPF can be performed with a low time requirement and still high concordance to the results of manual contouring. In contrast to short-axis views, the RV apex and especially the tricuspid valve can be well delineated in four-chamber views, so that the partly difficult contouring of the most basal slices of RV in short-axis views, which is error-prone for manual and automatic segmentation, can be circumvented. With high accuracy, the CPF method identified patients with reduced RV-EF ≤ 40%, which almost exactly represents the lower limit of normal RV-EF, which is indicated with 42% in men and a somewhat higher value of 46% in women, according to CMR reference values [8]. Purmah et al. (2021) showed, in a large CMR study, that patients with a RV-EF < 40% had a 3.1-fold higher risk for major adverse cardiovascular events [11].

The CPF method may be used as a rapid and simple screening tool for the initial RV assessment in different clinical scenarios. CPF may be helpful as a plausibility cross-check, especially for unexperienced readers, as well as in cases of difficult contouring or discrepancy between manual and automatic segmentation. Nevertheless, standard RV analysis by means of manual or automatic contouring should be part of each standardized interpretation of CMR images [9], particularly if the CPF method shows abnormal findings. Since CPF relies on a geometric assumption of the RV geometry being a truncated cone minus a truncated rhomboid pyramid, it cannot replace the standard analysis of the RV, where the whole RV is visualized regardless of its anatomy. Moreover, studies are warranted to prove if the CPF method is feasible in a broader spectrum of acquired and congenital RV diseases and if CPF has prognostic value.

### Limitations

The main limitations of this study are that this study was monocentric and performed on retrospective analyses in a relatively small cohort of consecutive patients, who were assigned to RV analysis. Moreover, the number of patients with abnormal RV size or function was limited, and patients with congenital heart disease and complex RV geometries were not included. Another limitation is that the right ventricular outflow tract (RVOT) is not included in the calculations of the CPF method. The RVOT accounts for up to 19% of total RV volume and up to 14% of total RV stroke volume [17], which can lead to incorrect measurements of RV volumes using the CPF method and might be particularly relevant for patients with pathological conditions of the RVOT. Moreover, RV-EF using the CPF method contains a calculation from two already calculated volumes, so that potential deviations can add up. This could further explain the discrepancies in RV-EF measurements. Furthermore, prognostic data have not been analyzed to assess the impact of the CPF method on patient outcomes. Therefore, further studies with an evaluation of clinical outcomes and with a larger sample size of patients with normal and abnormal right ventricular volumes and function are warranted to prove the results of this study and the efficacy of the CPF method in comparison to the standard method.

## 5. Conclusions

CMR-based calculations of RV volumes and function using the CPF method, which assumes a truncated cone and rhomboid pyramid model of RV anatomy, is feasible in all patients and only two linear measurements in four-chamber views are needed. The CPF method showed strong concordance to the standard CMR analysis and identified patients with enlarged RV size as well as reduced RV function with high accuracy.

## Figures and Tables

**Table 1:** Patients’ clinical characteristics and their left ventricular and right ventricular volumes and function.

Variables	*n* = 70
Age, years	56 ± 15
Male gender, *n* (%)	49 (70)
NYHA class	
I, *n* (%)	34 (49)
II, *n* (%)	27 (39)
III, *n* (%)	6 (9)
IV, *n* (%)	3 (4)
Coronary artery disease, *n*	25 (36%)
Pericarditis/myocarditis, *n*	8 (11%)
Cardiomyopathy, *n*	31 (44%)
Diabetes mellitus, *n*	9 (13%)
Arterial hypertension, *n*	28 (40%)
Dyslipidemia, *n*	19 (27%)
Smoking, *n*	26 (37%)
COPD/bronchial asthma, *n*	5 (7%)
CMR, standard method	
LV-EDV, mL	208.0 ± 101.5
LV-EDV index, mL/m^2^	104.2 ± 46.5
LV-ESV, mL	127.9 ± 94.9
LV-ESV index, mL/m^2^	63.9 ± 45.4
LV-EF, %	44.0 ± 16.2
RV-EDV, mL	162.6 ± 56.4
RV-EDV index, mL/m^2^	81.7 ± 24.1
RV-ESV, mL	89.4 ± 50.7
RV-ESV index, mL/m^2^	44.5 ± 23.2
RV-EF, %	48.4 ± 13.7
RV Dd, cm	4.2 ± 0.6
RV Ds, cm	3.5 ± 0.6
RV Ld, cm	7.7 ± 1.1
RV Ls, cm	6.5 ± 1.2

CMR: cardiovascular magnetic resonance; COPD: chronic obstructive pulmonary disease; Dd: diastolic basal diameter of the right ventricle at the level of the tricuspid valve; Ds: systolic basal diameter of the right ventricle at the level of the tricuspid valve; EDV: end-diastolic volume; EF: ejection fraction; ESV: end-systolic volume; Ld: diastolic baso-apical length from the mid part of the tricuspid valve to the right ventricular apex; Ls: systolic baso-apical length from the mid part of the tricuspid valve to the right ventricular apex; LV: left ventricular; NYHA: New York Heart Association; RV: right ventricular.

**Table 2:** Measurements of RV volumes and function using standard CMR analysis and the truncated cone and rhomboid pyramid model-based formula (CPF) method.

	Standard CMR Analysis	CPF-Based CMR Analysis	Difference	Correlation Coefficient r
RV-EDV index, mL/m^2^	81.7 ± 24.1	86.2 ± 29.4	−4.5 ± 19.0	0.765
RV-ESV index, mL/m^2^	44.5 ± 23.2	51.5 ± 22.5	−7.0 ± 14.4	0.801
RV-EF, %	48.4 ± 13.7	41.1 ± 13.5	7.2 ± 9.6	0.746

CMR: cardiovascular magnetic resonance; CPF: cone–pyramid formula; EDV: end-diastolic volume; EF: ejection fraction; ESV: end-systolic volume; RV: right ventricular.

**Table 3:** Intra- and interobserver variability of RV volumes and function using the cone–pyramid formula (CPF). Intra- and interobserver agreement of calculations of RV end-diastolic volume (RV-EDV), RV end-systolic volume (RV-ESV), and RV ejection fraction (RV-EF) using the CPF method in 30 randomly selected patients.

	Observer 1			Observer 2	
	CPF Analysis 1	CPF Analysis 2	IntraobserverICC with 95%-CI	CPF Observer 2	InterobserverICC with 95%-CI
RV-EDV, mL	171.2 ± 62.9	171.8 ± 64.1	0.9949 [0.9894–0.9976]	184.1 ± 65.2	0.9650 [0.9279–0.9832]
RV-ESV, mL	102.8 ± 44.8	101.1 ± 45.3	0.9959 [0.9915–0.9981]	94.3 ± 41.8	0.9574 [0.9126–0.9795]
RV-EF, %	40.3 ± 13.8	41.7 ± 12.6	0.9684 [0.9348–0.9849]	49.9 ± 11.1	0.7982 [0.6184–0.8986]

CI: confidence interval; CPF: cone–pyramid formula; EDV: end-diastolic volume; EF: ejection fraction; ESV: end-systolic volume; ICC: intraclass correlation coefficient; RV: right ventricular.

## Data Availability

The data presented in this study are available on request from the corresponding author. The data are not publicly available due to patient privacy.
